# A fully human anti-c-Kit monoclonal antibody 2G4 inhibits proliferation and degranulation of human mast cells

**DOI:** 10.1007/s11010-022-04557-3

**Published:** 2022-09-15

**Authors:** Kwang-Hyeok Kim, Jin-Ock Kim, Sang Gyu Park

**Affiliations:** College of Pharmacy, Ajou University, 206 World Cup-ro, Yeongtong-gu, Suwon, Gyeonggi-do 16499 Korea

**Keywords:** Mast cell, Mast cell disease, Allergy, c-Kit, Monoclonal antibody

## Abstract

Given that mast cells are pivotal contributors to allergic diseases, various allergy treatments have been developed to inhibit them. Omalizumab, an anti-immunoglobulin E antibody, is a representative therapy that can alleviate allergy symptoms by inhibiting mast cell degranulation. However, omalizumab cannot reduce the proliferation and accumulation of mast cells, which is a fundamental cause of allergic diseases. c-Kit is essential for the proliferation, survival, and differentiation of mast cells. Excessive c-Kit activation triggers various mast cell diseases, such as asthma, chronic spontaneous urticaria, and mastocytosis. Herein, we generated 2G4, an anti-c-Kit antibody, to develop a therapeutic agent for mast cell diseases. The therapeutic efficacy of 2G4 antibody was evaluated in LAD2, a human mast cell line. 2G4 antibody completely inhibited c-Kit signaling by blocking the binding of stem cell factor, known as the c-Kit ligand. Inhibition of c-Kit signaling led to the suppression of proliferation, migration, and degranulation in LAD2 cells. Moreover, 2G4 antibody suppressed the secretion of pro-inflammatory cytokines, including granulocyte–macrophage colony-stimulating factor, vascular endothelial growth factor, C–C motif chemokine ligand 2, brain-derived neurotrophic factor, and complement component C5/C5a, which can exacerbate allergy symptoms. Taken together, these results suggest that 2G4 antibody has potential as a novel therapeutic agent for mast cell diseases.

**Supplementary Information:**

The online version contains supplementary material available at 10.1007/s11010-022-04557-3.

## Introduction

c-Kit, a type III receptor tyrosine kinase, has five immunoglobulin-like domains (D1–D5) in the extracellular compartment and two kinase domains in the intracellular compartment [1, 2]. Stem cell factor (SCF), a ligand of c-Kit, binds to D1–D3, and D2 and D3 are important sites that determine the binding affinity of SCF with c-Kit [3]. SCF binding induces the homodimerization of c-Kit, which leads to phosphorylation. Phospho-c-Kit activates multiple signaling pathways, including phosphatidylinositol-3-kinase (PI3K)/Akt, mitogen-activated protein kinase (MAPK)/ERK, SRC, and Janus kinase (JAK)/signal transducer and activator of transcription (STAT) pathways, resulting in cell proliferation, survival, differentiation, and migration [2, 4, 5]. The SCF/c-Kit signaling plays an important role in hematopoiesis, and the expression and activation of c-Kit must be appropriately regulated during the differentiation process in hematopoietic cells [4]. Most early hematopoietic cells, including hematopoietic stem and progenitor cells, express c-Kit; however, the expression gets lost during differentiation and maturation, except for mast cells, eosinophils, and dendritic cells. Unlike other mature immune cells, mast cells highly express c-Kit even after differentiation [2]. SCF/c-Kit is essential for the growth, survival, and differentiation in mast cells, and also induces migration and invasion into specific tissues through SCF-chemotaxis [6–8]. Furthermore, SCF enhances the inflammatory response by synergistically increasing the degranulation and production of cytokines [9, 10].

Mast cells play a protective role in infectious disease against bacterial, viral, and parasitic infections through multiple mechanisms. Mast cells can recruit and stimulate immune cells, such as neutrophils, eosinophils, T cells, and dendritic cells, by releasing various cytokines. Proteases released by mast cells can break down pathogenic toxins [11]. Additionally, mast cells play a major role in allergic responses. Mast cells highly express the Fc epsilon receptor 1 (FcεRI), the receptor for immunoglobulin E (IgE). Allergen-specific IgE binds to FcεRI with high affinity (*K*_*D*_ = 10^−9^–10^−10^ M), and allergen-mediated cross-linking of IgE induces mast cell degranulation [12]. Degranulation results in the release of potent inflammatory mediators, such as histamine, proteases, cytokines, chemokines, and growth factors, which trigger allergic responses [11, 13, 14].

Despite the protective role of mast cells, excessive proliferation and abnormal activation cause various mast cell diseases. Allergic diseases, including asthma, chronic spontaneous urticaria (CSU), allergic rhinitis (AR), atopic dermatitis (AD), and anaphylaxis, are the most common mast cell diseases. Indeed, it has been found that the number of mast cell and the level of inflammatory mediator increase in the bronchi of patients with asthma. Asthma is a allergic disease that occurs in the lungs and airways due to an inordinate inflammatory response triggered by mast cells [15, 16]. Interestingly, the level of SCF significantly increases in asthmatic airways, leading to mast cell recruitment, proliferation, and survival [17]. In a previous study, it was demonstrated that SCF increased airway hyperreactivity, which was abrogated by SCF neutralization [18]. It suggests that the SCF/c-Kit signaling in mast cells closely contributes to asthma. Furthermore, in CSU, AR, and AD, the accumulation of mast cells and increased levels of inflammatory mediators released by mast cells are the main causes of these diseases [15, 19]. In addition to allergic diseases, mastocytosis and mast cell activation syndrome (MCAS) are also mast cell diseases. The major cause of mastocytosis is the expansion and accumulation of abnormal (neoplastic) mast cells in the skin (cutaneous) or other organs (systemic); it manifests various symptoms, such as itching, hives, vascular instability, headache, enlarged liver, and anaphylactic shock [20–22]. These abnormal mast cells mainly carry gain-of-function mutations of c-Kit, and c-Kit mutations were found in about 90% of patients with mastocytosis [22]. The causes and symptoms of MCAS are similar to those of systemic mastocytosis. MCAS is caused by abnormal and severe activation of mast cells, but no accumulation of mast cells in specific organs is observed [23, 24]. In addition, it is characterized by symptoms appearing in more than one organ, and symptoms appearing cyclically [25].

Most mast cell diseases are caused by IgE-mediated degranulation. Thus, anti-IgE monoclonal antibodies (e.g., omalizumab and ligelizumab), that inhibit mast cell degranulation, have been developed and approved by the Food and Drug Administration for the treatment of asthma and CSU. Omalizumab, the most representative anti-IgE antibody, is being used as a step five treatment for asthma with low efficacy against inhaled corticosteroids and long-acting β2 agonists [26]. Moreover, omalizumab is a third or fourth-line treatment for CSU patients who have developed resistance or suffer recurrence after receiving second-generation H1 antihistamines (e.g., cetirizine, desloratadine, ebastine, and emedastine) [27, 28]. The mechanism of action of omalizumab is to inhibit degranulation by neutralizing circulating IgE and reducing FcεRI expression. However, omalizumab has limitations that cannot reduce the number of mast cells, which can be the fundamental cause of mast cell diseases [29]. Therefore, repeated and continuous administration at 2 or 4 weeks interval is necessary for omalizumab treatment (subcutaneously 150–300 mg every 4 weeks or 225–375 mg every 2 weeks depending on IgE level and body weight) [30]. This is also the reason why omalizumab has insufficient efficacy in mastocytosis and MCAS [31–33].

To overcome the limitations of anti-IgE antibodies, we aimed to develop therapeutic antibody that can inhibit both the proliferation and degranulation of mast cells. Previous studies have demonstrated that 2G4 antibody, a fully human antibody, could inhibit the activation of SCF/c-Kit signaling in various cancer cell lines [34, 35]. In addition, another anti-c-Kit antibody 4C9 significantly reduced the expression of c-Kit [36]. Both 2G4 and 4C9 antibodies could bind to human c-Kit with high binding affinity (*K*_*D*_ of 2G4 = 2.83 × 10^−12^ M and *K*_*D*_ of 4C9 = 5.58 × 10^−9^ M) [34, 36]. In this study, we investigated whether 2G4 and 4C9 could inhibit the proliferation and degranulation in LAD2, a human mast cell line. Since excessive proliferation and abnormal activation cause mast cell diseases, the ultimate purpose of this study was to evaluate the potential for development of 2G4 and 4C9 antibodies as a treatment for mast cell diseases.

## Materials and methods

### Cell line and culture

LAD2 cell line was kindly provided by Dr. Kirshenbaum of the National Institutes of Health (Bethesda, MD, USA). LAD2 cells were cultured in StemPro-34 SFM (Thermo Fisher Scientific, MA, USA) with StemPro-34 nutrient supplement (2.5%, Thermo Fisher Scientific, MA, USA), l-glutamine (2 mM, Gibco, CA, USA), penicillin/streptomycin (1%, Hyclone, UT, USA), and recombinant human SCF (100 ng/mL, R&D Systems, MN, USA). Half of the medium was replaced weekly by adding an equal volume of fresh medium containing SCF. The cell density was maintained at 2–5 × 10^5^ cells/mL. The cells were incubated at 37 °C in 5% CO_2_ incubator.

### Flow cytometry analysis

To demonstrate antibody binding to c-Kit on the cell surface of LAD2 cells, flow cytometry assay was performed. LAD2 cells were starved of SCF for 24 h, because SCF can cause internalization and degradation of c-Kit. Cells were rinsed with phosphate-buffered saline (PBS) and blocked with PBS containing 5% bovine serum albumin (BSA) at 4 °C for 1 h. After blocking with BSA, Human BD Fc Block™ (2.5 μg/10^6^ cells, BD Biosciences, CA, USA) was treated to block binding of the antibody to the Fc receptor. The cells (2 × 10^5^ cells) were stained with 2G4, 4C9, or normal human IgG1 (Sino Biological, Beijing, China) at the indicated concentrations at 4 °C for 1 h. The cells were then rinsed thrice in PBS containing 2% BSA and stained with goat anti-human IgG secondary antibody (0.3 μg/mL, Invitrogen, CA, USA) at 4 °C for 1 h. After washing, the fluorescence signal was detected using CyFlow Cube6 (Sysmex Partec, Goerlitz, Germany), and the data analysis was performed using FCS Express 6 Flow (De Novo software, CA, USA).

### Western blot analysis

To verify whether 2G4 and 4C9 antibodies can inhibit SCF-mediated c-Kit activation, LAD2 cells were seeded into a 6-well culture plate (1 × 10^6^ cells/well) in SCF-deficient medium for 24 h. After SCF-starvation, LAD2 cells were pretreated with 2G4 or 4C9 antibody at the indicated concentrations at 37 °C for 1 h. The cells were then stimulated with 100 ng/mL of SCF for an additional 10 min. Thereafter, the cells were lysed in RIPA lysis buffer (pH 7.6, 20 mM Tris–HCl, 150 mM NaCl, 1 mM Na_2_EDTA, 1 mM EGTA, 1% NP-40, 1% sodium deoxycholate, 0.1% SDS, 10 mM β-glycerophosphate, 1 mM Na_3_OV_4_, 10 mM NaF, 1 μg/mL leupeptin, 1 mM PMSF, 5 μg/mL aprotinin, and 2 mM 2-mercaptoethanol). Phosphorylation of c-Kit and its downstream signaling molecules (Akt and Erk) was analyzed by Western blotting. The antibodies used herein were anti-phospho-c-Kit (Tyr 719, Tyr 823, Tyr568/570, and Tyr703, Cell Signaling Technology, MA, USA), anti-phospho Akt (Ser473, Cell Signaling Technology, MA, USA), anti-phospho-Erk1/2 (Cell Signaling Technology, MA, USA), anti-c-Kit (R&D Systems, MN, USA), anti-Akt (Santa Cruz Biotechnology, CA, USA), anti-Erk1/2 (Santa Cruz Biotechnology, CA, USA), and anti-α-tubulin (laboratory-made).

### Cell proliferation assay

LAD2 cells were seeded into 96-well culture plates (1 × 10^4^ cells/well), with or without 100 ng/mL of SCF. The cells were incubated with serial fivefold of 2G4, 4C9, or normal human IgG1 at a final concentration 100 μg/mL at 37 °C for 7 days. The cells were then stained with 10 μM Hoechst 33342 (Thermo Fisher scientific, MA, USA) for 30 min, and counted using Celigo imaging cytometer (Nexcelom, MA, USA).

### Migration assay

LAD2 cells were SCF-starved for 24 h. Thereafter, 1 × 10^6^ cells were seeded into the upper chamber of a 6-transwell plate with 8 μm pores (Costar, MA, USA) with low-supplement medium (StemPro-34 SFM with 0.5% StemPro-34 nutrient supplement, 2 mM l-glutamine, and 1% penicillin/streptomycin). Next, 1 μg/mL 2G4, 4C9, or normal human IgG1 was added to the upper chamber, and 100 ng/mL SCF was added to the lower chamber for 24 h. After removing the upper chamber, migrated cells in the lower chamber were microscopically counted using high-power field (HPF, ×40 magnification) in five different fields.

### Mast cell degranulation assay

To verify mast cell degranulation, a β-hexosaminidase release assay was carried out. To determine whether 2G4 or 4C9 increased degranulation, non-sensitized, IgE-sensitized, and interferon (IFN)-γ-sensitized LAD2 cells were prepared, respectively. Biotinylated human IgE (200 ng/mL, NBS-C Bioscience, Vienna, Austria) or IFN-γ (150 ng/mL, PeproTech, NJ, USA) was added to the cells at 37 °C for 24 h (with SCF-deficient medium). After sensitization, the cells were washed twice with HEPES buffer (10 mM HEPES, 137 mM NaCl, 2.7 mM KCl, 0.4 mM Na_2_HPO_4_, 5.6 mM glucose, 1.8 mM CaCl_2_, 1.3 mM MgSO_4_, and 0.04% BSA) and seeded onto a 96-well culture plate (10,000 cells/well). The cells were then incubated with 1 or 10 μg/mL 2G4, 4C9, or normal human IgG1 at 37 °C (without CO_2_) for 1 h. Streptavidin (2 ng/mL, Sigma-Aldrich, MO, USA), as a positive control, was used to crosslink biotinylated-IgE. After centrifugation at 450×*g* for 5 min, 50 μL of supernatant was added to 100 μL *p*-nitrophenyl *N*-acetyl-β-d-glucosamide (PNAG) solution (3.5 mg/mL, Sigma-Aldrich, MO, USA) at 37 °C (without CO_2_) for 1.5 h. When 50 μL of glycine buffer (0.4 M) was added, the yellow color indicated β-hexosaminidase activity [37]. Absorbance at 405 nm wavelength was measured using SPECTROstar Nano microplate reader (BMG Labtech, Ortenberg, Germany).

To examine whether 2G4 or 4C9 could suppress degranulation synergistically increased by SCF, IgE-sensitized LAD2 cells were resuspended in HEPES buffer and seeded onto a 96-well culture plate (10,000 cells/well). Cells were pretreated with 2G4, 4C9, or normal human IgG1 at the indicated concentrations at 37 °C for 0.5 h, and treated with 100 ng/mL of SCF for an additional 0.5 h. Thereafter, streptavidin was added to crosslink biotinylated-IgE for 0.5 h, and β-hexosaminidase release assay was carried out.

### Cytokine-release assay

LAD2 cells (1 × 10^6^ cells/mL) were sensitized with biotinylated-IgE (200 ng/mL) at 37 °C for 24 h. The cells were then washed twice with PBS and seeded into a 12-well culture plates (1 × 10^6^ cells/well) with low-supplement medium. Thereafter, 2G4 antibody (1 μg/mL), SCF (100 ng/mL), and streptavidin (10 ng/mL) were added to the cells at intervals of 0.5 h. After 24 h of incubation, the cell-culture supernatant was harvested, and the cytokines released by LAD2 cells were blotted using the Human XL cytokine array kit (R&D Systems, MN, USA) according to the manufacturer’s protocol. The intensity of each blot was measured using the ImageJ software (US National Institutes of Health, MD, USA).

### Statistical analysis

Graphing and statistical analyses were performed using GraphPad Prism 5 (GraphPad Software, CA, USA). *P* values were measured by two-tailed Student’s *t* test or one-way ANOVA with Dunnett’s post-test. Statistical significance was set at *P* < 0.05.

## Results

### 2G4 antibody inhibits SCF/c-Kit signaling

**Fig. 1:**
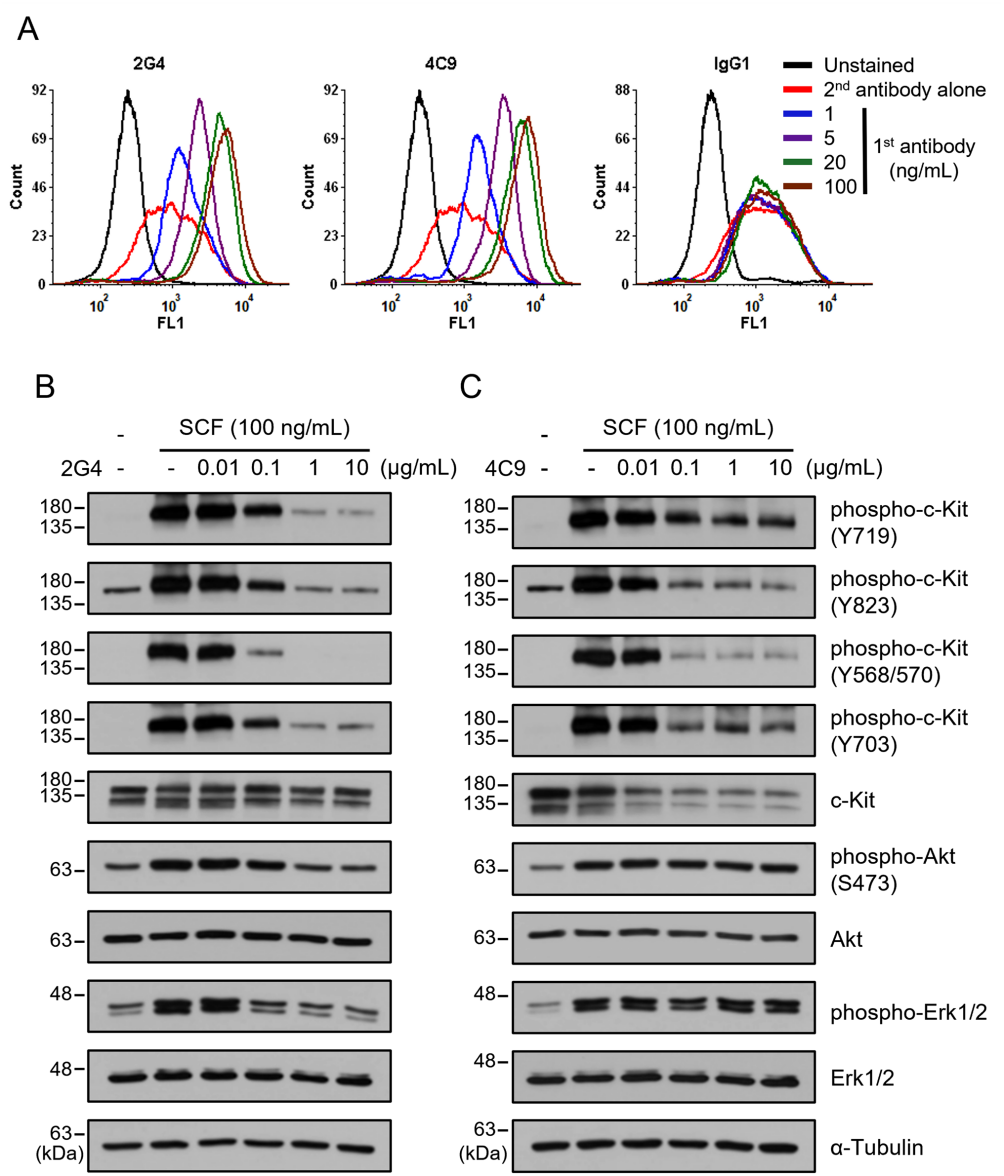
2G4 and 4C9 antibodies bind to c-Kit and inhibit c-Kit activation in LAD2 cells. **A** LAD2 cells were incubated with 2G4, 4C9, or normal human IgG1 at the indicated concentrations for 1 h. After washing, FITC-conjugated secondary antibody was added for 1 h. The fluorescence was detected by flow cytometry. **B**, **C** SCF-starved LAD2 cells were incubated with 2G4 antibody (**B**) or 4C9 antibody (**C**) at the indicated concentration for 1 h. Thereafter, the cells were stimulated by 100 ng/mL of SCF for an additional 10 min. Phosphorylation of c-Kit, Akt, and Erk1/2 was analyzed by Western blotting. α-Tubulin was used as a loading control

In previous studies, we demonstrated that 2G4 and 4C9 antibodies bind to c-Kit with high binding affinity (*K*_*D*_ of 2G4 = 2.83 × 10^−12^ M and *K*_*D*_ of 4C9 = 5.58 × 10^−9^ M). Moreover, 2G4 antibody, as an antagonist of c-Kit, inhibited SCF/c-Kit signaling in various cancer cell lines [34, 35]. In this study, the human mast cell line LAD2, which highly expresses FcεRI and c-Kit (Supplementary Fig. 1), was used for the experiments. LAD2 cells were treated with Human BD Fc Block™ to inhibit non-specific Fc receptor-mediated binding, and antibody binding to c-Kit on the cell surface was analyzed using flow cytometry. Consequently, both 2G4 and 4C9 antibodies bound to LAD2 cells in a dose-dependent manner, and the fluorescence signal was saturated at a concentration of 100 ng/mL (Fig. 1A). However, the binding signal of normal human IgG1 did not increase, except for the non-specific signal by the secondary antibody.

Thereafter, we investigated whether 2G4 or 4C9 antibody inhibit c-Kit activation induced by SCF in LAD2 cells. Phosphorylation of c-Kit and its downstream signals, Akt and Erk1/2, was significantly increased by 100 ng/mL SCF, and the phosphorylation was maintained for up to 60 min (Supplementary Fig. 2). Pre-treatment with 2G4 antibody inhibited c-Kit phosphorylation induced by SCF in a dose-dependent manner (Fig. 1B). 2G4 antibody inhibited phosphorylation at all tested tyrosine residues of c-Kit (Y719, Y823, Y568/570, and Y703). In addition, phosphorylation of Akt and Erk1/2 decreased by 2G4 antibody in a dose-dependent manner. While 4C9 antibody also inhibited c-Kit phosphorylation, it did not inhibit the phosphorylation of Akt and Erk1/2 at all (Fig. 1C). Interestingly, 4C9 antibody significantly downregulated the total c-Kit expression in a dose-dependent manner. Therefore, the decrease in phospho-c-Kit might be due to the downregulation of total c-Kit expression.

### 2G4 antibody suppresses cell proliferation and migration of mast cells

**Fig. 2:**
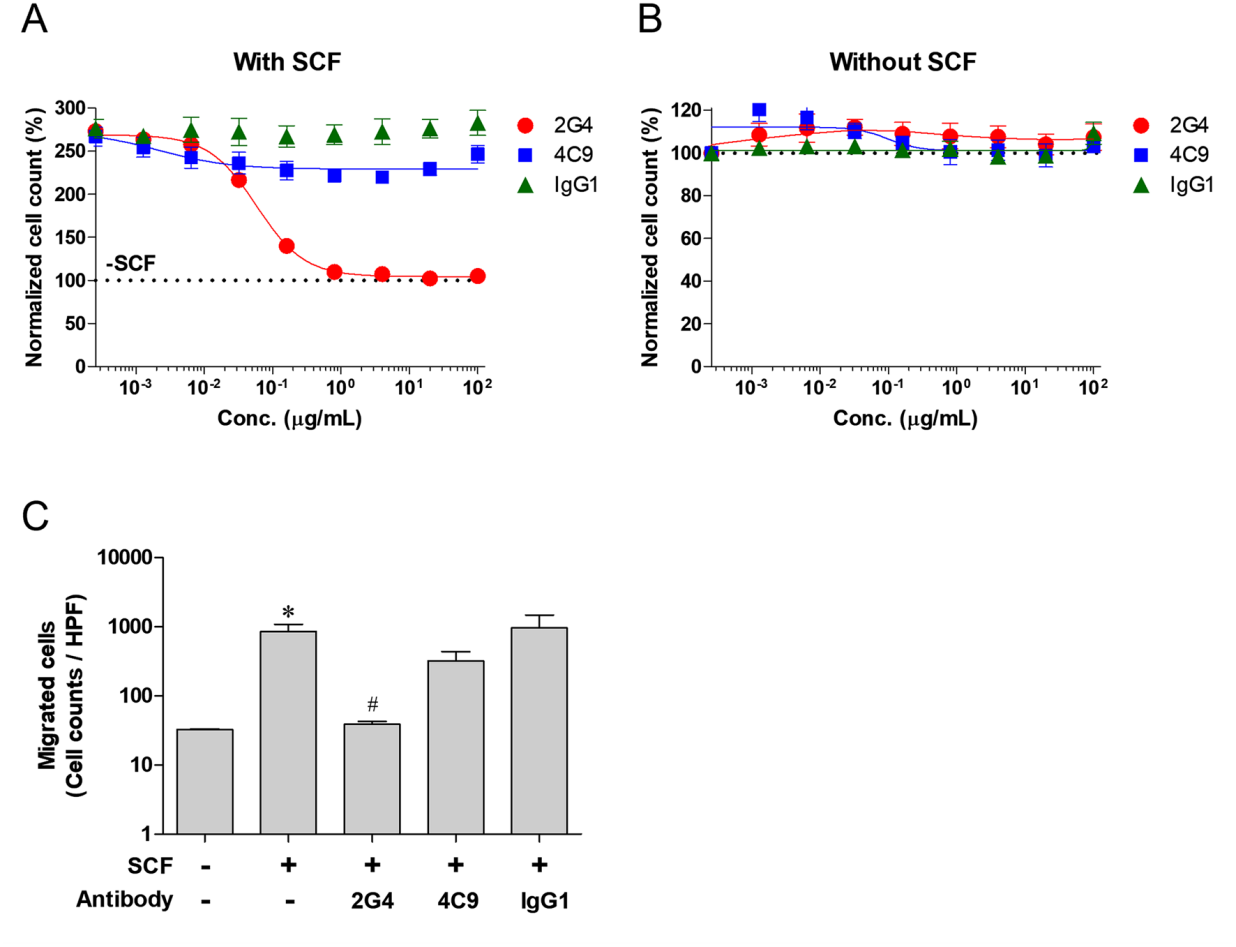
2G4 antibody inhibits cell proliferation and migration in LAD2 cells. **A**, **B** LAD2 cells were incubated with 2G4, 4C9, or normal human IgG1 at the indicated concentrations in culture medium with SCF (**A**) or without SCF (**B**) for 7 days. Thereafter, cells were stained with 10 μM Hoechst 33342 and counted using a Celigo Imaging Cytometer. The black dashed line (100%) indicates normalized cell counts in the well without SCF and antibodies at 7 days. **C** Migration assay was carried out in a 6-transwell plate with 8 μm pores. LAD2 cells (1 × 10^6^ cells) and the antibodies (1 μg/mL) were added into the upper chamber and SCF (100 ng/mL) was added into the lower chamber for 24 h. Migrated cells in the lower chamber were microscopically counted using a HPF in five different fields. All results represent the mean ± SD of three independent experiments. * vs. SCF^−^/Antibody^−^ and # vs. SCF^+^/Antibody^−^. *P < 0.05, and ^#^*P* < 0.05 (Student’s two-tailed *t* test)

In the cell proliferation assay, the number of LAD2 cells was increased by 100 ng/mL SCF by more than 2.5-fold for 7 days. SCF-mediated proliferation was suppressed by 2G4 antibody in a dose-dependent manner, but not by 4C9 or normal human IgG1 (Fig. 2A). The half-maximal inhibitory concentration (IC_50_) value of 2G4 antibody against LAD2 cells was 0.058 μg/mL, and the proliferation was completely inhibited by 2G4 antibody at concentrations above 0.8 μg/mL. In the absence of SCF, LAD2 cells did not proliferate, and 2G4, 4C9, and normal human IgG1 had no effect on cell proliferation at all (Fig. 2B). This implied that 2G4 antibody does not directly induce cell death against LAD2 but can inhibit proliferation mediated by SCF.

In the migration assay using a transwell plate with 8 μm pore polycarbonate membrane, the number of cells passing through the membrane increased more than 26-fold by 100 ng/mL of SCF (Fig. 2C). The increase in migration induced by SCF was completely suppressed by 1 μg/mL of 2G4 antibody at the basal level. In contrast, LAD2 migration was partially inhibited by 4C9 antibody, but no significant differences were observed (*P* = 0.1071). There was no decrease in migration with the presence of normal human IgG1.

### 2G4 antibody inhibits mast cell degranulation synergistically increased by SCF

**Fig. 3:**
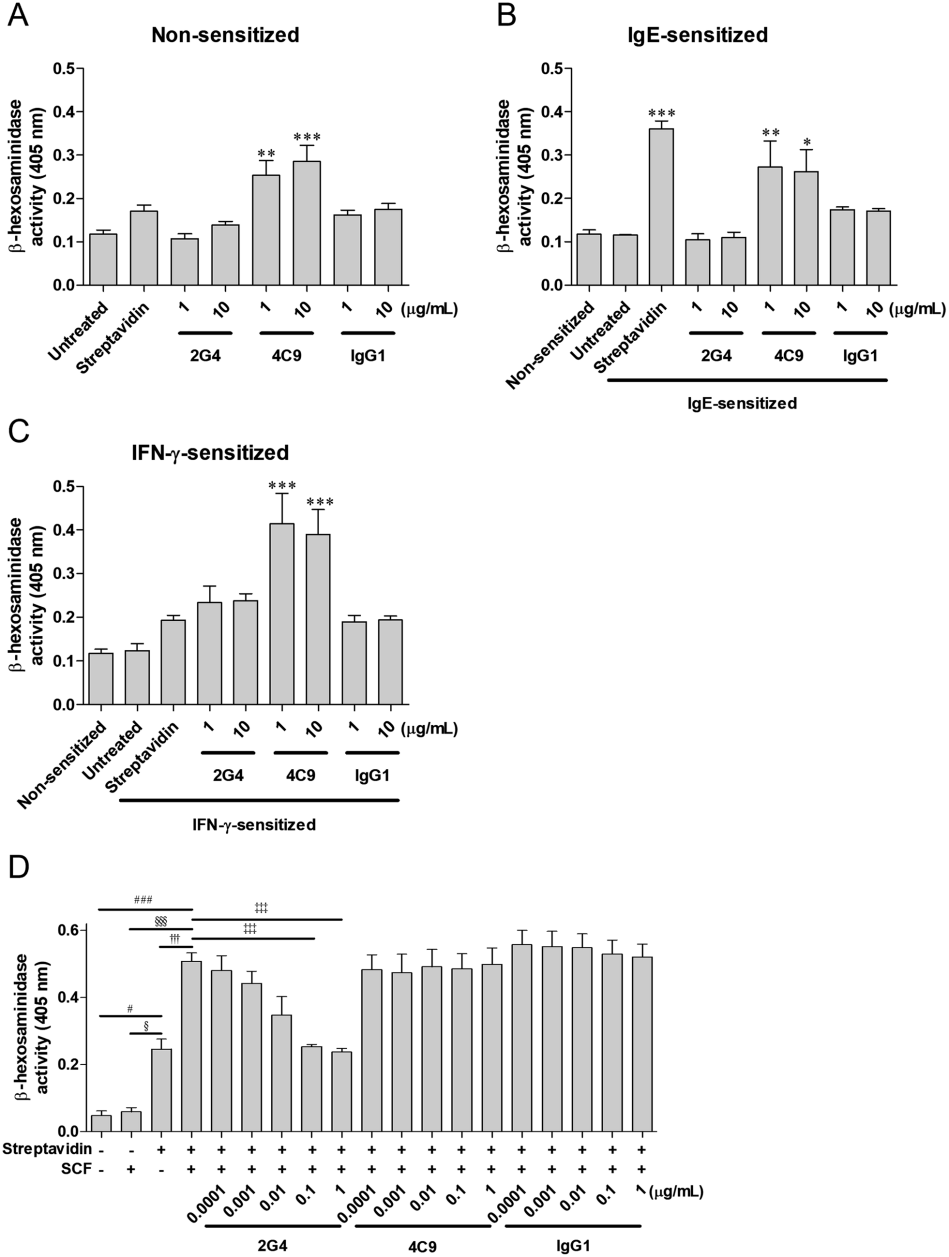
2G4 antibody inhibits IgE-mediated degranulation enhanced by SCF. **A** LAD2 cells were SCF-starved for 24 h. The cells were then incubated with 2G4, 4C9, normal human IgG1, or streptavidin for 1 h, and β-hexosaminidase release assay was performed. **B**, **C** LAD2 cells were sensitized with biotinylated human IgE (**B**) or IFN-γ (**C**) for 24 h in SCF-deficient medium. The cells were then incubated with 2G4, 4C9, normal human IgG1 or streptavidin for 1 h. Following this, β-hexosaminidase release assay was carried out. Streptavidin was used as a positive control to crosslink biotinylated-IgE. **D** LAD2 cells were SCF-starved and sensitized with biotinylated human IgE for 24 h. Cells were treated with antibodies (2G4, 4C9, or normal human IgG1), SCF (100 ng/mL), and streptavidin (2 ng/mL) in sequence at 30 min intervals. After 30 min of streptavidin treatment, β-hexosaminidase release assay was performed to analyze the degranulation of LAD2. All results represent the mean ± SD of three independent experiments. *, **, and *** vs Untreated, # vs. SCF^−^/Streptavidin^−^, § vs. SCF^+^/Streptavidin^−^, † vs. SCF^−^/Streptavidin^+^, and ‡ vs. SCF^+^/Streptavidin^+^. *P < 0.05, **P < 0.01, ***P < 0.001, ^#^*P* < 0.05, ^##^*P* < 0.01, ^###^*P* < 0.001, ^§^*P* < 0.05, ^§§^*P* < 0.01, ^§§§^*P* < 0.001, ^†^*P* < 0.05, ^††^*P* < 0.01, ^†††^*P* < 0.001, ^‡^*P* < 0.05, ^‡‡^*P* < 0.01, and ^‡‡‡^*P* < 0.001 (one-way ANOVA with Dunnett’s post-test)

Human mast cells express Fc gamma receptors (FcγRs), including FcγRI (CD64) and FcγRII (CD32), but not FcγRIII (CD16) [38]. Binding of the Fc region of IgG to FcγRs may trigger degranulation which can cause hypersensitivity reactions (HSRs) in patients receiving therapeutic antibodies. Moreover, multimeric IgG-antigen immune complexes can bind to FcγRs with high-avidity interactions [39–41]. Therefore, we performed β-hexosaminidase assay to determine whether 2G4 or 4C9 antibody increased degranulation in LAD2 cells (Fig. 3A–C). There was no significant increase in degranulation mediated by 2G4 antibody and normal human IgG1 in non-sensitized LAD2 cells, but 4C9 antibody increased degranulation in a dose-dependent manner, compared with untreated cells (Fig. 3A). In IgE-sensitized cells, 2G4 antibody did not increase degranulation, whereas 4C9 antibody significantly increased degranulation (Fig. 3B). Normal human IgG1 slightly increased degranulation; however, the difference was not statistically significant (*P* > 0.5808). Streptavidin for IgE ligation as a mimetics of allergen was used as a positive control to demonstrate activity of mast cell degranulation. Streptavidin increased the degranulation more than threefold compared to untreated cells (Fig. 3B). It has been reported that IFN-γ strongly increases the expression of FcγRI in human mast cells, and degranulation may be enhanced by FcγRI [40, 42, 43]. In IFN-γ-sensitized cells, 2G4 antibody (*P* > 0.1784) and normal human IgG1 (*P* > 0.6074) slightly increased degranulation, but the difference was not statistically significant (Fig. 3C). Meanwhile, 4C9 antibody significantly increased the degranulation, which was higher than degranulation in non- or IgE-sensitized cells.

Although SCF alone is insufficient to induce mast cell degranulation, SCF synergistically increases degranulation with allergen-specific IgE [44]. In this study, SCF alone did not increase degranulation, but SCF synergistically increased IgE-mediated degranulation by more than twofold (Fig. 3D, Supplementary Fig. 3B). The increase in degranulation was inhibited by 2G4 antibody in a dose-dependent manner (IC_50_ = 0.00636 μg/mL), and the degranulation decreased up to the level of streptavidin^+^/SCF^−^ at the concentrations above 0.1 μg/mL. 4C9 antibody and normal human IgG1 did not inhibit degranulation at all. Therefore, this result indicated that 2G4 antibody can inhibit the excessive increase in degranulation by SCF. In contrast, all 2G4, 4C9, and normal human IgG1 did not inhibit degranulation induced by IgE or SCF alone (Supplementary Fig. 3).

### 2G4 antibody inhibited SCF-mediated modulation of cytokine secretion by mast cells

**Fig. 4:**
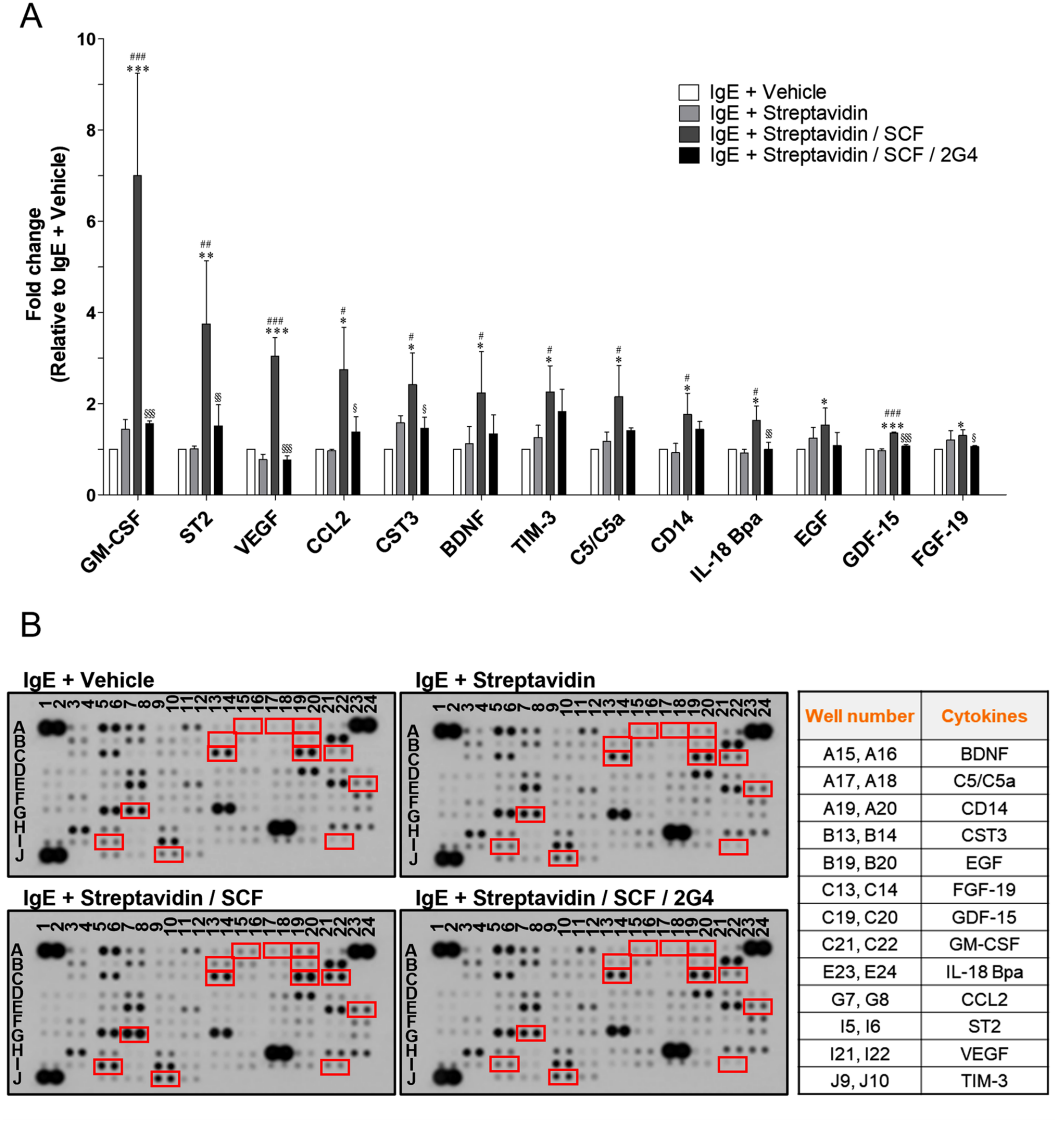
2G4 antibody inhibits the modulation of cytokine secretion. **A** LAD2 cells were sensitized with biotinylated-IgE in SCF-deficient medium for 24 h. Then, 2G4 antibody (1 μg/mL), SCF (100 ng/mL), and streptavidin (10 ng/mL) were added to the LAD2 cells sequentially at 30 min intervals. After 24 h, cytokine release was analyzed using a Human cytokine array kit. The result represents the mean ± SD of three independent experiments. **B** Representative blot images of the cytokine array. Cytokines indicated in the graphs are highlighted by red squares. *, **, and *** vs. IgE + Vehicle, #, ##, and ### vs IgE + Streptavidin, §, §§, and §§§ vs. IgE + Streptavidin/SCF. *P < 0.05, **P < 0.01, and ***P < 0.001, ^#^*P* < 0.05, ^##^*P* < 0.01, and ^###^*P* < 0.001, ^§^*P* < 0.05, ^§§^*P* < 0.01, and ^§§§^*P* < 0.001 (Student’s two-tailed *t* test)

Mast cells secrete various cytokines that recruit and stimulate various immune cells. Therefore, the modulation of cytokine secretion by mast cells can augment the inflammatory response, and increased inflammatory response can be a serious factor that aggravates symptoms in allergy patients [45, 46]. Herein, we found that various cytokines significantly increased following treatment with 100 ng/mL of SCF (Fig. 4). The increase in the level of these cytokines was potently inhibited by treatment with 1 μg/mL of 2G4 antibody. In particular, granulocyte–macrophage colony-stimulating factor (GM-CSF) is increased by SCF by more than sevenfold, but the increase is completely reduced by 2G4 antibody to the basal level. In addition, suppression of tumorigenicity 2 (ST2), vascular endothelial growth factor (VEGF), C–C motif chemokine ligand 2 (CCL2, also known MCP-1), cystatin C (CST3), brain-derived neurotrophic factor (BDNF), T cell immunoglobulin and mucin-domain containing-3 (TIM-3), and complement component C5/C5a increased more than twofold, but all of these cytokines were effectively reduced by 2G4 antibody. Conversely, a few cytokines, including CCL5, macrophage colony-stimulating factor (M-CSF), and interleukin-2 (IL-2), were downregulated following treatment with SCF (Supplementary Fig. 4). The downregulation was also suppressed by 2G4 antibody. The cytokines shown in Fig. 4 and Supplementary Fig. 4 (highlighted with red squares) were statistically different in three repeated experiments. Certain cytokines which seem to show differences in representative blotting images but not highlighted (e.g., A7, A8, A11, A12, G13, and G14) did not show statistical significance, so they were not graphed. Collectively, SCF induces the modulation of cytokine secretion by mast cell, and the modulation is mainly shown by an increase in pro-inflammatory cytokines that exacerbate mast cell diseases. 2G4 antibody inhibited the modulation of cytokines and demonstrated its feasibility as a therapeutic antibody for the treatment of mast cell diseases.

## Discussion

The fundamental cause of mast cell diseases is an increase in the number of mast cells in the inflamed site. Currently, various therapeutic agents for allergic diseases, including antihistamines, corticosteroids, and anti-IgE antibodies, effectively alleviated symptoms; however, they could not decrease the number of mast cells. To overcome this limitation, innovative therapeutic strategies need to be developed. Therefore, we developed an antibody that could inhibit both proliferation and degranulation in mast cells. In this study, we investigated whether 2G4 or 4C9 antibody can inhibit growth and function in LAD2 cells and considered the feasibility of treatment for mast cell diseases. LAD2 is SCF-dependent human mast cell line with wild-type c-Kit. Therefore, LAD2 cells are activated and proliferate in an SCF-dependent manner, like primary mast cells. Additionally, LAD2 expresses FcεRI, FcγRI, histamine, and tryptase, and is degranulated by the cross-linking of FcεRI or FcγRI, leading to the release of inflammatory mediators. Therefore, LAD2 is a suitable cell line for mast cell experiments [47].

2G4 and 4C9 antibodies have different characteristics for blocking the ligand. 2G4 antibody binds to the D2/D3 of c-Kit which broadly overlaps with binding site of SCF [34]. Furthermore, 2G4 antibody binds to c-Kit with high binding affinity (*K*_*D*_ = 2.83 × 10^−12^ M), which is more than 500-fold stronger than that between SCF and c-Kit (*K*_*D*_ = 1.5 × 10^−9^–3 × 10^−10^ M) [34, 48–50]. Therefore, 2G4 antibody acts as a ligand blocker to inhibit SCF binding. In this study, 2G4 antibody effectively inhibited the activation of c-Kit signaling induced by SCF in LAD2 cells (Fig. 1B). In addition, inhibition of SCF/c-Kit signaling by 2G4 antibody led to complete inhibition of proliferation (Fig. 2A). Conversely, SCF binding sites for c-Kit have minimal overlap with epitope of 4C9 antibody, so 4C9 antibody could not block binding of SCF [36]. In addition, given that the binding affinity of 4C9 antibody (*K*_*D*_ = 5.58 × 10^−9^) was similar to the affinity of SCF, 4C9 antibody could not suppress the binding between SCF and c-Kit. However, our previous studies have revealed that 4C9 antibody has characteristics in which 4C9 antibody induces c-Kit internalization and degradation within a short period of time [36]. In this study, 4C9 antibody dose-dependently decreased the level of total c-Kit for 1 h in LAD2 cells (Fig. 1C). Although 4C9 antibody decreased the level of phospho-c-Kit as well as that of the total c-Kit, 4C9 antibody could not reduce that of phospho-Akt and phospho-Erk1/2, which resulted in failure to inhibit proliferation of LAD2 cells (Figs. 1C, 2A).

Inhibition of proliferation and migration of mast cells has substantial implications in the treatment of mast cell diseases because an increase in the number of mast cells in organs or tissues is an underlying cause. Increase in the number of mast cells has been observed in various mast cell diseases, such as asthma, CSU, anaphylaxis, and mast cell leukemia [15, 20, 23, 51–53]. An increase in mast cells in the airway induces increased inflammation and immune remodeling. The immune remodeling leads the recruitment of various inflammatory cells, such as eosinophils, basophils, and helper T cells, leading to further exacerbation of the symptoms [18, 26]. In addition, given that multifocal dense infiltrates of mast cells (> 15 mast cells in aggregates) in bone marrow or other tissue (commonly skin), biopsies is one of the major criteria in the diagnosis of mastocytosis, it is evident that accumulation of mast cells is a major cause of mastocytosis [52, 54]. In this study, 2G4 antibody potently inhibited the proliferation and migration of LAD2 cells (Fig. 2). The mechanism of action governing inhibition of mast cell proliferation and migration is a unique advantage of 2G4 antibody, which differs from conventional therapeutics, including antihistamines, corticosteroids, and anti-IgE antibodies.

2G4 antibody potently inhibits degranulation and modulation of cytokine production in mast cells. In this study, SCF significantly increased the degranulation and secretion of various cytokines, including GM-CSF, ST2, VEGF, CCL2, BDNF, and C5/C5a (Figs. 3D, 4). These cytokines play a critical role in allergic responses. It is known that GM-CSF is essential for the development, function, and survival of eosinophils. Increased GM-CSF levels in lesions of patients with asthma and CSU induce eosinophil recruitment and survival, resulting in excessive accumulation of eosinophils [55–57]. Eosinophils, as well as mast cells, are considered therapeutic targets for allergic diseases because they play a critical role in chronic and severe symptomatology [58, 59]. Eosinophils produce and secrete fibrogenic factors, such as fibroblast growth factor (FGF), heparin binding epidermal growth factor, IL-4, IL-13, IL-17, nerve growth factor, platelet derived growth factor, and transforming growth factor-β (TGF-β), leading to the development of severe asthma [60, 61]. Therefore, inhibition of GM-CSF secretion from mast cells by 2G4 antibody is expected to show a synergistic activity against allergic diseases by suppressing eosinophil recruitment and survival. Interestingly, c-Kit is also expressed in eosinophils, and it is reported that the activation of SCF/c-Kit signaling in eosinophils enhances the expression of FGF-5, FGF-7, and TGF-β [62]. Therefore, in addition to reducing the recruitment of eosinophils through mast cell inactivation, 2G4 antibody may directly exhibit therapeutic effects by inhibiting the SCF/c-Kit signal in eosinophils. However, the inhibitory effect of 2G4 antibody against eosinophils should be investigated in further studies. In addition, other cytokines whose secretion is increased by SCF can also exacerbate allergic response. VEGF promotes the migration of inflammatory cells by increasing vascular permeability, and CCL2 promotes the recruitment of various inflammatory cells through chemotaxis [63–65]. BDNF induces bronchoconstriction by promoting the proliferation of airway smooth muscle cells in asthmatic patients, and C5/C5a contributes to the pathological features of asthma, such as mucus release, contraction of smooth muscle cells, increased vascular permeability, and infiltration of inflammatory cells [66, 67]. Conversely, a few cytokines, including IL-2, which can cause an anti-inflammatory response, are reduced by SCF (Supplementary Fig. 4). IL-2 promotes the function and survival of regulatory T cells (Tregs), which play a role in preventing allergic diseases, such as AR and AD, by regulating immune homeostasis [68, 69]. The IL-2 downregulation induced by SCF reduces the activity of Treg, thereby increasing the inflammatory response. Therefore, 2G4 antibody can enhance Treg activation by increasing the anti-inflammatory cytokine IL-2. Taken together, SCF can modulate cytokines secretion by mast cell and the modulation mainly shown by an increase in various pro-inflammatory cytokines and a decrease a small number of anti-inflammatory cytokines; however, 2G4 antibody exhibits therapeutic efficacy against mast cell diseases by inhibiting SCF-mediated cytokine modulation, which accelerates the progression and symptoms.

Drug administration can cause HSRs. In particular, therapeutic antibodies may induce mast cell degranulation via FcγRs, which are upregulated in human mast cells, and cross-linking of FcγRs by IgG induces degranulation, similar to FcεRI [39, 70]. LOP628, an anti-c-Kit antibody–drug conjugate developed by Novartis, failed in a phase I clinical trial due to HSR. This is because the immune complex formed from c-Kit-LOP628 increased FcγRI-mediated degranulation of human mast cells [40]. It is reported that the large immune complex composed of IgG and antigen binds to FcγRs with high avidity and can induce FcγR cross-linking, leading to degranulation [41]. In addition, given that IFN-γ enhances expression of FcγRI and FcγRI-mediated degranulation, it could be particularly fatal in allergy patients [40, 43]. To surmount this drawback, we designed anti-c-Kit antibodies to have no effector function mediated by FcγRs [34, 35]. Moreover, we had to evaluate whether 2G4 or 4C9 antibody did not increase mast cell degranulation in vitro. As a result, 2G4 antibody alone did not increase mast cell degranulation, even in the presence of IgE or IFN-γ (Fig. 3A–C). In contrast, 4C9 antibody significantly increased the degranulation under all tested conditions. Although the detailed mechanism by which 4C9 antibody induces degranulation needs to be elucidated through further studies, it cannot be ignored that the c-Kit-4C9 antibody complex may induce FcγR-mediated degranulation like LOP628. Therefore, to develop a therapeutic agent using 4C9 antibody, further antibody engineering is required to lower FcγR-mediated degranulation. In addition, we examined whether 2G4 antibody can induce cytotoxicity of normal cell (line), including COS7, HUVEC, PBMC, MS-1. However, 2G4-mediated cytotoxicity was not observed up to 200 μg/mL (results not shown). In vivo repeated toxicity study using normal C57BL/6 mice (q1w × 3) did not show any body weight change up to 40 mg/kg (results not shown).

In summary, our study shows that 2G4 antibody has potential as a therapeutic agent for mast cell diseases, with a mechanism different from that of conventional therapeutics. 2G4 antibody binds to c-Kit with high affinity and completely blocks the binding of SCF, a ligand of c-Kit. Blockade of SCF/c-Kit signaling effectively inhibits cell proliferation, migration, degranulation, and cytokine release in human mast cells. Therefore, these results suggest that 2G4 antibody has potential as a therapeutic agent for mast cell diseases.

## Supplementary Information

Below is the link to the electronic supplementary material.

Supplementary file1 (PDF 447 KB)

## Data Availability

The datasets generated and/or analyzed during the current study are available from the corresponding author upon reasonable request.
