# Supplementary material for: A fully human anti-c-Kit monoclonal antibody 2G4 inhibits proliferation and degranulation of human mast cells
Source: Mol Cell Biochem. 2022 Sep 15;478(4):861–73. doi: 10.1007/s11010-022-04557-3 (PMC10066129; doi:10.1007/s11010-022-04557-3)
Supplement: Supplementary file 1 — Supplementary file1 (PDF 447 KB) [file 11010_2022_4557_MOESM1_ESM.pdf]

Supplementary Figures

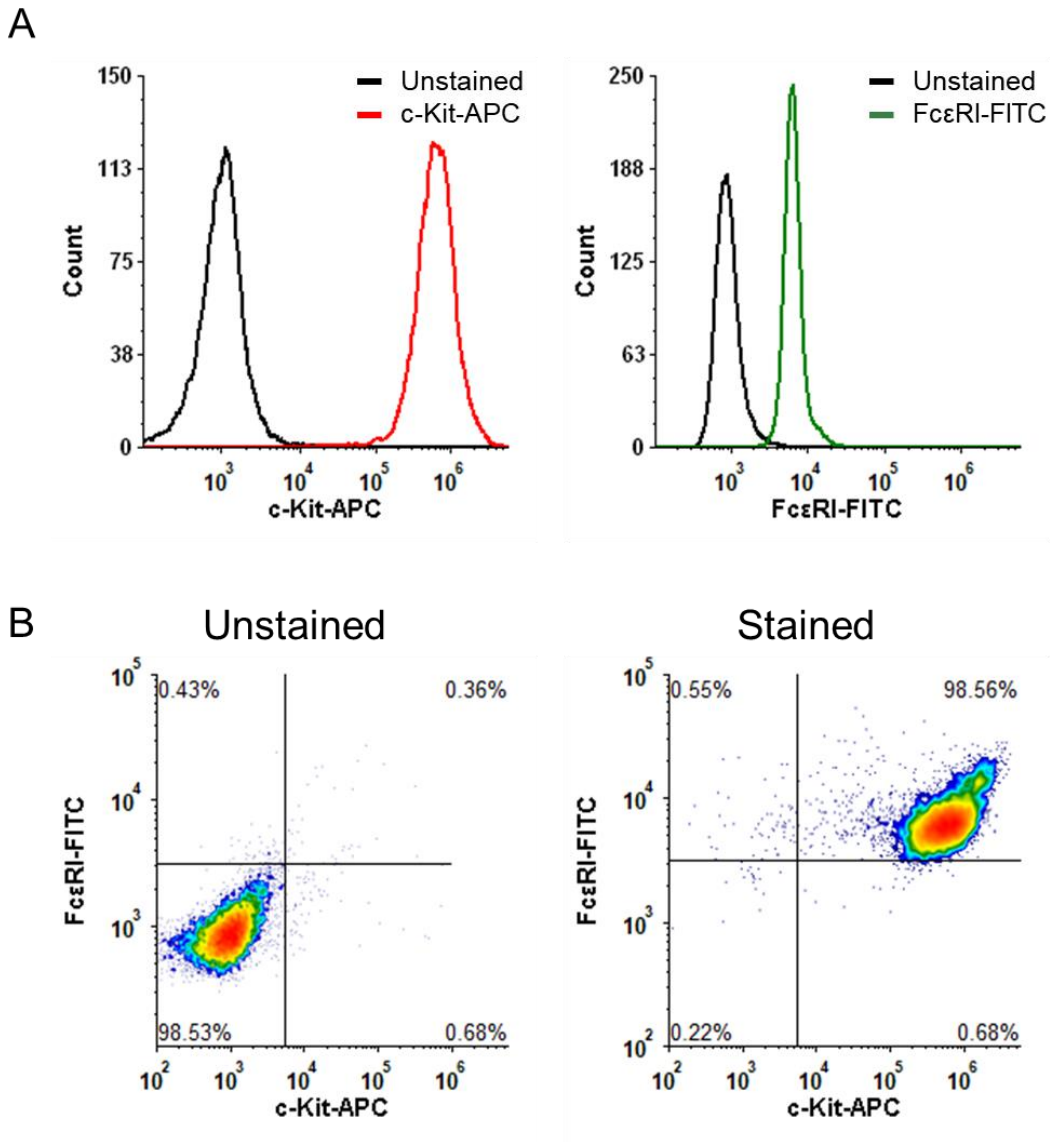

Supplementary Fig. 1 Expression of c-Kit and FcεRI in LAD2 cells. Flow cytometry analysis was performed to confirm the expression of c-Kit and FcεRI in LAD2 cells. The cells (2×10^5 cells) were stained with APC-conjugated anti-c-Kit antibody and FITC-conjugated anti-FcεRI antibody at 4 °C for 1 h. After washing, the fluorescence was detected using CyFlow Cube6. The expression of c-Kit and FcεRI is shown in histograms **(A)** and density plots **(B)**

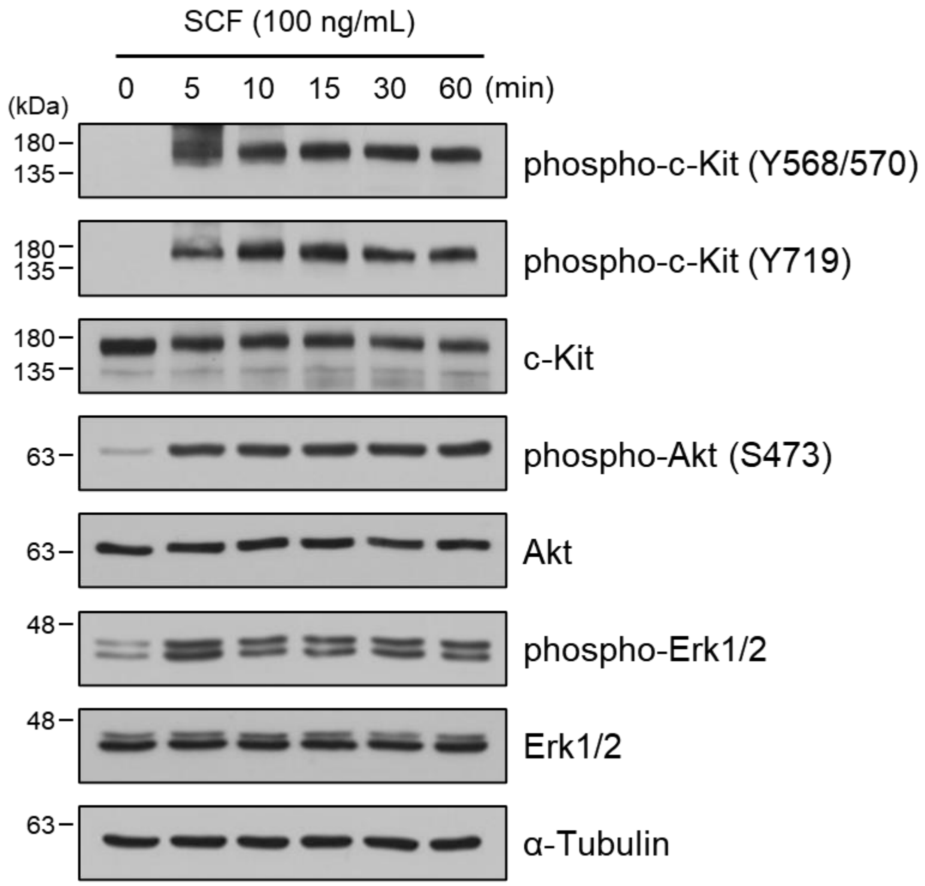

Supplementary Fig. 2 SCF activates c-Kit signaling in LAD2 cells. LAD2 cells were SCF-starved for 24 h. The cells were then stimulated by SCF (100 ng/mL) for the indicated time. Phosphorylation of c-Kit, Akt and Erk1/2 was analyzed by western blotting. α-Tubulin was used as a loading control

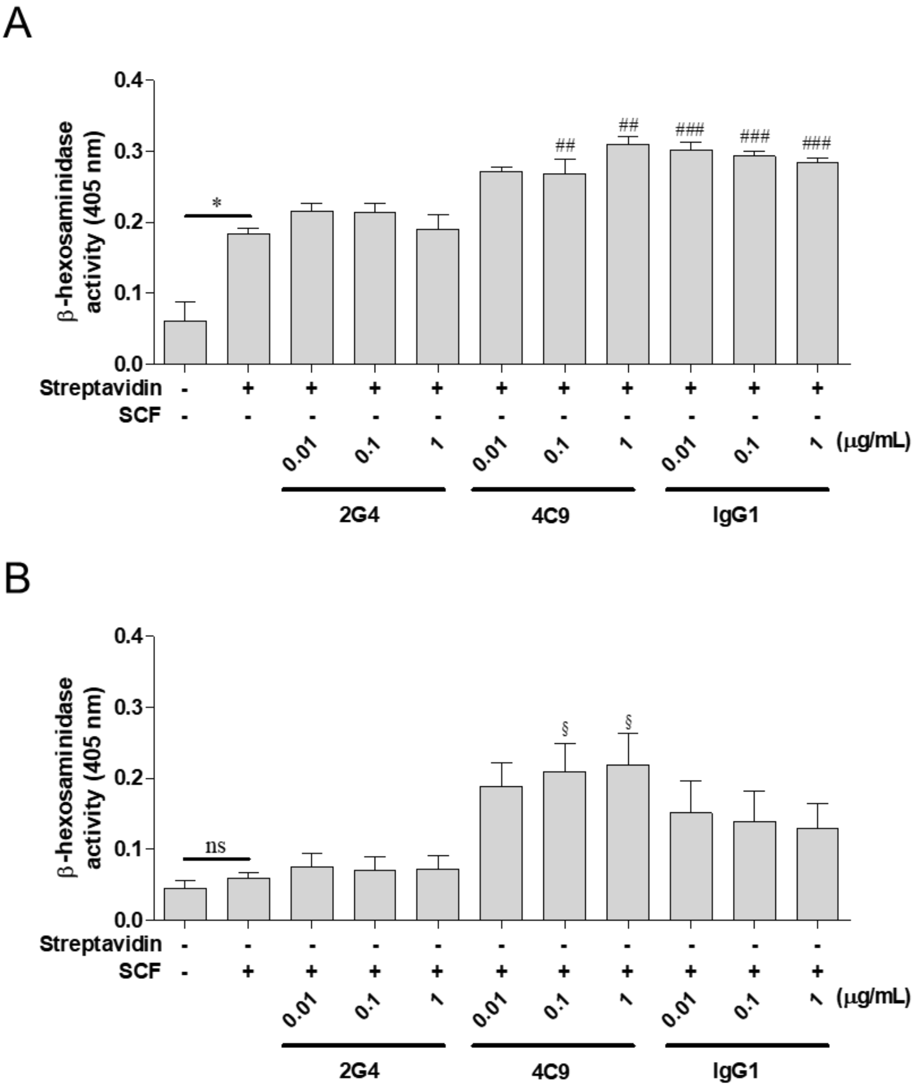

Supplementary Fig. 3 2G4 and 4C9 antibodies did not inhibit degranulation mediated by IgE or SCF alone. **A,** LAD2 cells were SCF-starved and sensitized with biotinylated-IgE for 24 h. Antibodies (2G4, 4C9, or normal human IgG1), and streptavidin (2 ng/mL) were treated to the cells at intervals of 30 min. After 30 min of streptavidin treatment, β-hexosaminidase release assay was performed to analyze the degranulation. **B,** LAD2 cells were SCF-starved for 24 h. Antibodies and SCF (100 ng/mL) were treated to the cells at intervals of 30 min. After 30 min of SCF treatment, β-hexosaminidase was measured. All results represent the mean ± SD of three independent experiments. *, **, and *** vs. SCF$^-$/Streptavidin$^-$, #, ##, and ### vs. SCF$^-$/Streptavidin$^+$, §, §§, and §§§ vs. SCF$^+$/Streptavidin$^-$. * $P < 0.05$, ** $P < 0.01$, *** $P < 0.001$, # $P < 0.05$, ## $P < 0.01$, ### $P < 0.001$, § $P < 0.05$, §§ $P < 0.01$, §§§ $P < 0.001$ (one-way ANOVA with Dunnett's post-test)

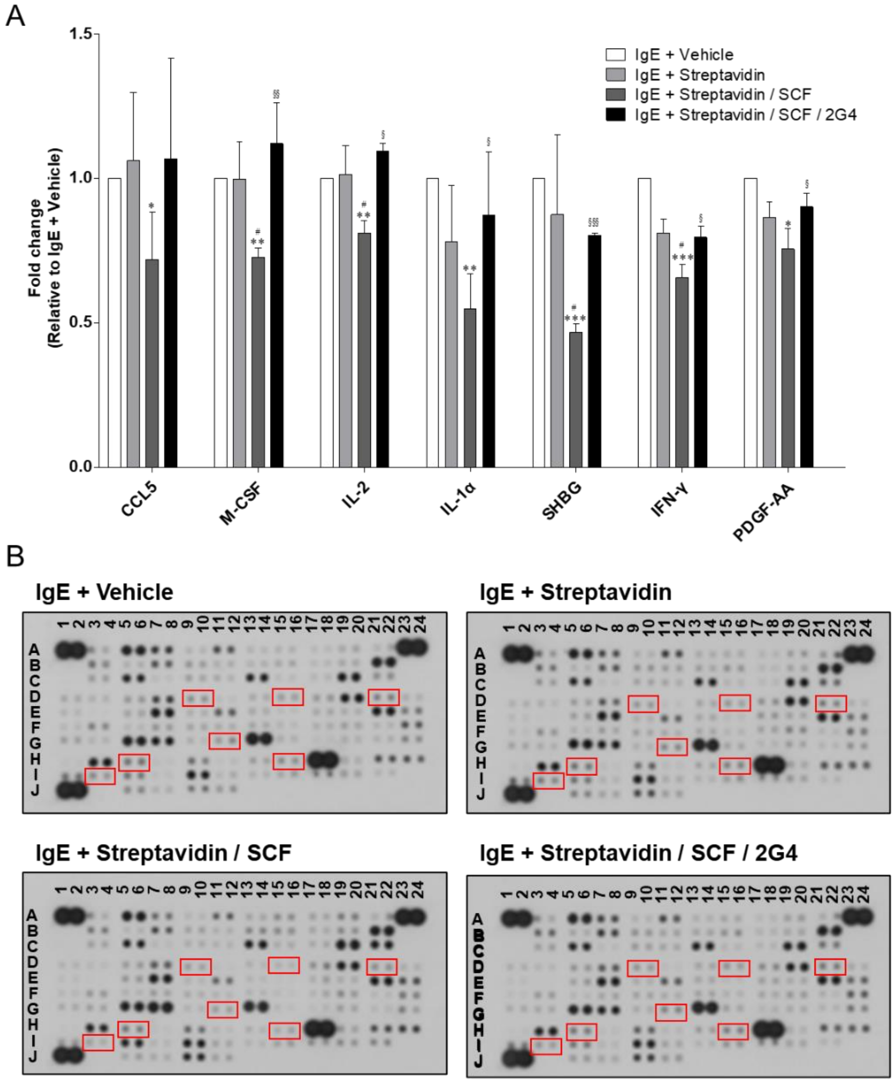

Well number	Cytokines
D9, D10	IFN-γ
D15, D16	IL-1α
D21, D22	IL-2
G11, G12	M-CSF
H5, H6	PDGF-AA
H15, H16	CCL5
I3, I4	SHBG

Supplementary Fig. 4 2G4 antibody inhibits modulation of cytokines secretion. **A,** Certain cytokines, including CCL5, M-CSF, and IL-2, were decreased by treatment with SCF (100 ng/mL). And the decrease in cytokine secretion was inhibited by 2G4 antibody. The result represents the mean ± SD of three independent experiments. **B,** Representative blot images of cytokine array. Cytokines which indicated in the graphs are highlighted by red squares. *, **, and *** vs. IgE + Vehicle, #, ##, and ### vs. IgE + Streptavidin, §, §§, and §§§ vs. IgE + Streptavidin/SCF. * $P < 0.05$, ** $P < 0.01$, and *** $P < 0.001$, # $P < 0.05$, ## $P < 0.01$, and ### $P < 0.001$, § $P < 0.05$, §§ $P < 0.01$, and §§§ $P < 0.001$ (Student's two-tailed *t*-test)
